# Weak General but No Specific Habituation in Anticipating Stimuli of Presumed Negative and Positive Valence by Weaned Piglets

**DOI:** 10.3390/ani8090149

**Published:** 2018-08-22

**Authors:** Angela Henzen, Lorenz Gygax

**Affiliations:** 1 Centre for Proper Housing of Ruminants and Pigs, Federal Food Safety and Veterinary Office FSVO, Agroscope, Tänikon, CH-8356 Ettenhausen, Switzerland; angela.henzen@tierschutz.com; 2 Animal Behaviour, Department of Evolutionary Biology and Environmental Studies, University of Zurich, Winterthurerstrasse 190, CH-8057 Zurich, Switzerland; 3 Animal Husbandry, Albrecht Daniel Thaer-Institute of Agricultural and Horticultural Sciences, Humboldt-Universität zu Berlin, Philippstraße 13, D-10115 Berlin, Germany

**Keywords:** pigs, habituation, valence, stimuli

## Abstract

**Simple Summary:**

Everyday experience shows that getting used to negative events is difficult, whereas positive events are quickly taken for granted. This is relevant for basic questions on how we and other animals deal with emotional events. It has also practical implications because we would like to avoid negative events as much as possible and increase the positive events for animals in our care. Here, we repeatedly treated pairs of piglets with a variety of presumed negative, intermediate and positive events and measured their restlessness as well as the reaction of the autonomic nervous system, while the piglets expected these events. We found limited evidence that the reactions of the piglets changed in a systematic way with more repetitions of the different events. Both our experimental set-up and measurements have been used in similar ways before. Therefore, it is likely that we did not find a consistent pattern because we had not correctly assessed the negativity-positivity of the events used or their intensity from the point of view of the piglets.

**Abstract:**

Positive and negative stimuli have asymmetric fitness consequences. Whereas, a missed opportunity may be compensated, an unattended threat can be fatal. This is why it has been hypothesised that habituation to positive stimuli is fast while it may be difficult to habituate to negative stimuli, at least for primary (innate) stimuli. However, learning of secondary stimuli may delay the process of habituation. Here, we tested 64 weaned piglets in pairs. In three phases, lasting one week each, piglets were exposed five times to a stimulus of presumed negative, intermediate, or positive valence. Etho-physiological measurements of heart rate, heart rate variability, and general movement activity were collected during the last 4 min before the confrontation with the stimulus (anticipation phase). We found no consistent effect of the interaction between the valence of the stimuli and the repetition and a main effect of valence on our outcome variables. Therefore, we could neither support the hypothesis that piglets habituate more slowly to secondary positive stimuli than to primary negative stimuli nor that they habituate less to primary negative stimuli when compared with other stimuli. These results could have been caused because stimuli may not have differed in the presumed way, the experimental design may not have been adequate, or the measures were not suitable for detecting habituation to the stimuli. Based on the stimuli used here and their valence that was only presumed, we could not support the hypothesis that the habituation process differs according to the valence of the stimuli.

## 1. Introduction

Emotional stimuli are likely to shape behavioural decisions. But how should a subject react to the same emotional stimulus when encountered repeatedly? Should the reactions be adjusted, that is should a subject habituate to such stimuli or should the reactions be kept strong? Nettle [1] (p. 32) describes this issue as follows: “… The negative emotion can be very persistent. It is possible to imagine living in chronic fear, if the sources of fear are not remedied. On the other hand, you might be joyful when a long-lost cousin arrives, but you can’t imagine remaining joyful for as long as he stays. The joy gradually ebbs away, even if the bringer of joy is still present …”

This is an illustration for the potential temporal effects that may result from the asymmetry in fitness consequences of stimuli with different valence (i.e., differing on an axis ranging from negative to positive). Negative stimuli may have far-reaching detrimental and irreversible consequences, and they therefore need to be addressed with high priority. In general, persistent or repeated negative stimuli should consequently not lead to habituation. In the specific case and if experience shows that the effect of such a repeated, potentially negative stimulus may not be as consequential as expected, habituation may occur to save on potentially costly (in terms of time and energy) reactions. Indeed, it has been found that the habituation to many stimuli depends on the initial stimulus strength and frequency of exposure in that habituation is slower with strong stimuli and faster with more exposures [2]. With positive stimuli, habituation seems inherent, such that these stimuli do not pre-occupy attention with the side-effect that negative stimuli might be missed [1]. Many positive stimuli may not be perceived as such when first encountered. Subjects may need to learn that such stimuli have positive consequences [3]. In these cases, it may take longer until habituation sets in. The positive stimulus is considered to be a secondary (learnt) emotional stimulus [4], whereas Nettle’s [1] conjecture focuses on primary emotional stimuli (innate, [4]). The asymmetry in fitness consequences leads also to the hypothesis that there are more primary negative than primary positive stimuli because it is more crucial for a subject to address potentially detrimental (and fatal) stimuli than missing out on (some) opportunities. Therefore, more positive stimuli may be secondary and need to be learnt. Given these considerations, there is a basic interest in whether and how an organism can and does habituate to stimuli of different valence.

Habituation implies that an initial behavioural or physiological reaction to a stimulus is dampened until it can no longer be distinguished from baseline [2]. Changes in etho-physiological indicator variables may occur in frequency and magnitude. Stronger intensity of a stimulus or a higher frequency of exposure may slow or accelerate the habituation process, respectively [2]. For example, it was found that humans reduce their initially prolonged reaction time quickly if they face repeated emotional distractors [5]. Moreover, physiological reactions and reports of the valence of one’s own feelings habituated in humans, whereas a cognitive assessment (conceptual knowledge) of the valence of the stimuli did not [6]. In pigs, [7] found conditioned differences in etho-physiological measures, even after repeated exposure to presumed emotional stimuli, though they did not investigate how this change occurred over time.

The valence of the stimuli is linked to the emotion that the stimulus elicits in a (mammalian) subject when is confronted with any given stimulus [8,9]. Emotions are defined often as short-term affective reactions and include concerted physiological, behavioural, and cognitive responses that occur when confronted with a specific stimulus or a situation [10,11]. They can be viewed as the mechanism resulting from an evolutionary selection process that helps an animal to assess situations in respect to fitness threats and opportunities and are therefore essential for survival [12]. In this sense, emotions signal relevance of situations and events to an organism [4,13].

In animals, it is generally assumed that isolation, that is separation from conspecifics (e.g., [14,15,16,17]), feed frustration (e.g., [18,19,20]), as well as sudden and loud stimuli (e.g., [7], fear: [21,22]) are perceived as negative and are accordingly used in experiments. To elicit positive situations [23], feed related situations are mostly used (e.g., [7,24]). Some authors have also used gentle human-animal interactions (e.g., [25,26,27,28]) or reunions with conspecifics (e.g., [17]). Indicator variables are often used to assess the affective reactions in animals. General movement activity (e.g., [29,30]) and reactions in the autonomic nervous system (e.g., [7,31,32]), as reflected by heart rate variables, provide such indicators that can be used to compare affective reactions in a broad range of experimental situations.

With the recent increase of interest in animal affective states and the testing of such states, the question of habituation to positive and negative stimuli also has relevance in respect to experimental design. The effect of a stimulus may wear off quickly due to habituation and lose its effect. In contrast, novelty can interfere with other characteristics of a stimulus, such as its valence. For example, a novel positive stimulus might elicit a state similar to human surprise. The etho-physiological instantiation of this state may be more similar to a negative reaction. Therefore, it may be difficult to assess valence of stimuli if they also differ in familiarity. Some habituation to novel stimuli is therefore desirable to disentangle the effects of novelty and valence.

The habituation process to emotional stimuli is also of practical relevance in respect to animal welfare. Whereas, a quick habituation to negative stimuli would be desirable to reduce negative circumstances such as e.g., handling by a veterinarian, lasting positive stimuli are sought to lighten up intensive housing conditions [33]. Given the outline at the start of this introduction, this may indeed be difficult to achieve.

Here, we investigated in weaned piglets whether the differences in habituation speed to a broad variety of stimuli occurred according to their presumed valence. Pairs of piglets were repeatedly exposed to with a presumed negative, intermediate, and positive stimulus. The same stimulus was used within the pairs but a variety of stimuli were used for the different pairs. We used a variety of stimuli to ensure that our measurements depended on the valence of the stimuli rather than on the specific type of stimulus that we investigated. It can be assumed that the negative stimuli used here had characteristics that are perceived immediately as negative and that the positive stimuli included novel aspects that needed some experience for being assessed. In this sense, it is likely that the negative stimuli can be considered primary and the positive stimuli secondary. The piglets’ general activity, heart rate, and heart rate variability was measured during the anticipation of the stimuli [34]. We chose the anticipation phase for our measurements because activity in that phase is likely to be comparable among the different stimuli with which the piglets were confronted though the specific behaviour, even during anticipation, may be more specific [35]. We expected that habituation, reflected by a return to baseline values would occur faster for presumed negative stimuli in comparison with presumed positive stimuli. This was because we expected the consequences of the presumed negative stimuli to be apparent with the first confrontation whereas the piglets might have needed to make some repeated experience with the outcome of the presumed positive stimuli before perceiving their value. For the intermediate stimuli, we did not expect a strong initial reaction, and, therefore, only little margin for habituation.

## 2. Materials and Methods

### 2.1. Animals

64 Swiss Large White piglets were used in this experiment. The piglets were born at Agroscope Tänikon, Switzerland. Until the age of one month (±7 days), the piglets were housed together with their mother in a free farrowing pen (7 m^2^). After this, they were weaned and moved to a pen with a deep-litter area and nest boxes that were covered by a roof of an open-front barn (so-called “Kooman pen”; 17.2 m^2^). In this pen, several litters of similar age were combined in groups of up to 50 piglets. The piglets remained in the Kooman pen for one month (±7 days), i.e., during the experimental period. The complete housing group was fed ad-libitum in these pens. Therefore, our experimental pigs were not food deprived.

When single piglets were exposed to the experimental situation during pilot trials, they were very active and highly vocal. Because such reactions are interpreted as signs of stress, as found specifically during social separation [36,37,38,39], piglets were confronted with the experimental stimuli in pairs in the final experiment. 

To avoid issues of etho-physiological differences between the sexes as well as potential similarities due to genetic relatedness, only pairs that were composed of female piglets from different litters were used in this experiment. Within a litter, 2–4 female piglets were chosen randomly. 

The Veterinary Office of the Canton Thurgau approved the experiment and its procedures (application: TG 03/14).

### 2.2. Experimental Design

The experiment was conducted between February and June 2015. Each piglet pair went through three experimental phases lasting one week each. In a given week, the piglets were confronted daily with the same stimulus for a short duration in an experimental pen (one daily “trial”). Stimuli were varied between weeks and differed in the presumed valence (negative, intermediate, positive). Each experimental phase lasted five days followed by a break of two days. The order of the stimulus valence across the three weeks was balanced between piglet pairs, so that all the possible variations of order were used equally often to avoid systematic bias due to carry-over effects. This also meant that the piglets’ reactions were balanced in respect to their age.

For each presumed valence, several different types of stimuli were used but only one of these was used with any given pair of piglets. We considered the following stimuli as positively valenced stimuli: a scuff area made from sawdust with hidden popcorn (40 × 80 cm^2^, 30 cm deep), a feed ball stuffed with hay hanging from the ceiling (diameter 30 cm, 30 cm above ground made of wire mesh), compressed food that could be chewed (cylinder shaped, 20 × 30 cm^2^), and two balls from which food items dropped if moved (plastic ball, diameter 20 cm). With all of these stimuli, the food reward was not immediately perceivable and at least some interactions with these stimuli were necessary to experience their rewarding nature. This is why we assumed that some learning of these stimuli was necessary and why we considered these stimuli as secondary in our hypotheses.

A small amount of openly accessible feed (seven pieces of popcorn on solid ground) and a novel, non-manipulable object (piece of wood, 1 × 5 × 120 cm^3^) were considered as intermediately valenced stimuli. Possibly, both of these stimuli were slightly positive because the first included food and the second was manipulable to a certain extent. Nevertheless, the stimuli with presumed positive valence included additional aspects, for example scuffing or moving a ball with the snout, which were assumed to make those stimuli more positive.

The negatively valenced stimuli included the separation of both piglets from each other (isolation; one piglet remained in the start box, the other was introduced to the empty experimental pen, see below), waving plastic bags fixed to a broomstick at the piglets for 10 s every 30 s, the presentation of inaccessible feed in a closed plexiglas box (7 × 19 × 24 cm^3^), and popping a total of six balloons every 30 s. The threatening or fear-eliciting effect of these stimuli (with exception of the inaccessible feed) should have been immediately perceivable by the piglets. Nevertheless, they could potentially learn that the treatment stopped after a relatively short time and that nothing seriously bad would happen to them. Therefore, we considered these stimuli in our hypotheses as primary stimuli to which the piglets might nevertheless habituate.

The pairs of piglets were confronted with the different stimuli for up to 10 min per trial. To keep the overall strain on the piglets during the experiment low, the trials were ended if the pairs showed clear and repeated signs of stress (increase in activity and vocal behaviour [36,37,38,39]). Nevertheless, the presumed negative stimuli were obviously perceived as stressful by the pigs because the trials were regularly stopped after they showed these signs of stress. Early trial ends were more common during the first week and during confrontations to a negatively valenced stimulus (waving plastic bags, inaccessible feed, popping of balloons). These trials lasted 5–7 min. Moreover, situations with a piglet in isolation were very stressful and usually stopped in the second minute.

All the possible combinations of the four negative, two intermediate, and four positive stimuli were randomly assigned to the different pairs of piglets resulting in 32 combinations for the 32 pairs. In addition, all the permutations of the sequences of negative, intermediate, and positive stimuli were used. That is, each piglet pair was tested with a different combination of stimuli that had been randomly assigned. The 32 pairs of piglets were tested in four batches. The number of piglet pairs per batch depended on the number of unrelated female piglets that were available in the litters. Five piglet pairs participated in the first batch whereas nine pairs were included in the second, third, and fourth batch.

**Figure 1:**
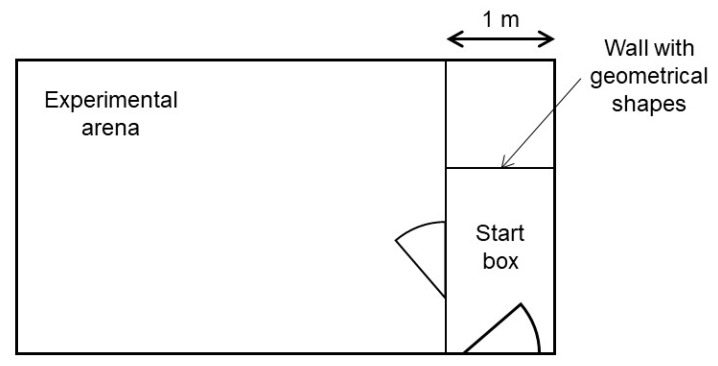
Set-up of the experimental pen.

All confrontations with the stimuli took place in an experimental pen. This pen was located approximately 50 m from the Kooman pen in another compartment of the barn without other animals present. It consisted of a start box and an experimental arena connected by a door (Figure 1). In every trial, one of the long start box walls was marked with one of three different geometrical shapes (black triangle, green rectangle, blue circle). These geometrical shapes indicated the valence of the stimulus, to which the piglets would be confronted to in the experimental arena. All the possible shape-valence combinations were balanced across piglet pairs. The idea of the shapes was to ease the learning of the piglets of what they were about to face, and therefore reduce carry over effects between the weeks. For our experiment, it was sufficient, though, if the piglets learnt the different situations without using the information of these shapes. The experimenter controlled both doors.

### 2.3. Experimental Procedure: Training

Day-3 to 0 refer to the four days before the first trial of each pair. In the morning of day-3, piglets were weaned and marked with an earmark. To identify experimental piglets in the Kooman pen more easily, they received a second earmark of a different colour. In the morning of day-2, the experimental piglets were all marked additionally with different patterns painted on their back (RAIDEX, Dettingen an der Erms, Germany) for clear visual identification in the group and the experimental pen. Then, all of the piglets used in one batch could explore the passage from the Kooman pen to the experimental pen as well as the experimental pen for 2 h. During that period, the piglets were constantly rewarded with popcorn lying on the ground (1–2 popcorn/min). However, popcorn was not provided in the room where the experimental pen was situated and within the experimental pen in order to keep it as neutral as possible. In the afternoon of day-2, piglets repeated the same procedure in pairs for 15 min. This time, they were additionally equipped with the measurement devices (see below). On day-1 and 0, the piglets stayed in the Kooman pen without experimental interference. 

### 2.4. Experimental Procedure: Testing

**Figure 2:**
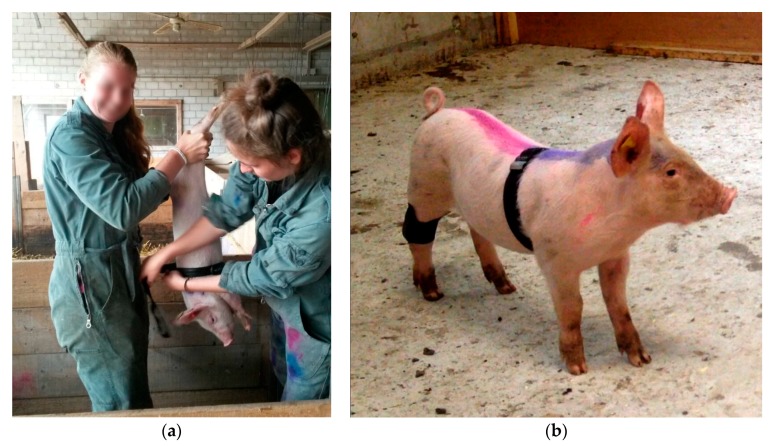
Experimental equipment: (**a**) a piglet being equipped; (**b**) a piglet pair equipped with an MSR data logger on the right hind leg to measure the general movement activity and a polar belt around the breast to measure RR intervals (interbeat interval) (captured from a video).

Piglets were caught singly by gripping one of their hind legs in the Kooman pen. Catching a piglet required 30 to 60 s. During approximately 30 s, the piglet was held upside-down to attach the measurement devices (Figure 2). While the second piglet of a pair was equipped, the first, fully equipped piglet had free access to the passage leading to the experimental pen. Although this access was free, the first piglet waited, without exception, within a radius of 20 m for the second piglet. Once the piglet pair was reunited, they walked together to the experimental pen and into the start box. In only 1–2 trials per batch, the piglet pair had to be driven into the start box (using a board of 1 × 1.5 m^2^). As soon as piglets were equipped with the measurement devices, they did no longer show any signs of disturbance. Overall, this indicated that the procedure was mildly stressful to the pigs and that they recovered quickly.

The piglets were kept in the start box for 4 min before the door to the experimental arena was opened and where they were confronted with a stimulus. At the end of the trial, the piglets voluntarily walked back to the Kooman pen, where the measurement devices were taken off.

### 2.5. Measurements 

Pilot experiments had shown that the different stimuli, both within and between valence, elicited different behaviours and different levels of activity in the experimental arena. These were, of course, informative of the specific effect of the stimuli on the piglets. It was not possible, though, to define indicators that would be comparable across the stimuli of the same valence. Therefore, any measurements taken in the experimental arena during exposure to a stimulus would not have been comparable between the different stimuli in respect to their valence. That is why the focus of the quantitative data collection was on the period when the piglets were in the start box (anticipation phase). 

During the pilot trials, it was observed that the piglets showed increased activity during the first min of the anticipation phase in the start box. We therefore excluded the first minute of the 4 min of data collection in the start box from data analysis.

#### 2.5.1. Heart Rate and Heart Rate Variability Measurement 

RR intervals (interbeat interval) were measured non-invasively with the Polar Team^2^ system (Polar Electro Oy, Kempele, Finland), a heart rate monitoring system developed for humans. Two electrodes integrated in the belt registered the time between heartbeats. To increase the conductivity, Signacreme^®^ (Parker Laboratories, Fairfield, CT, USA) electrode cream was applied between the electrodes and the skin. The belt was fixed, so that the electrodes were located to the left side of the abdomen, around the breast, and behind the forelegs. The data that were collected during the trial were saved in a Polar Logger (Polar Electro Oy, Kempele, Finland), which was attached to the belt and connected through two snap-fasteners with the integrated electrodes. Each evening, after the trials, the data were transmitted to a computer via a serial interface (Polar Team^2^, version 1.4.3) and then analysed with the Polar ProTrainer 5 (Equine Edition, version 5.10.121, Polar Electro Oy, Kempele, Finland). This program was used to detect one or two 1-min sequences per trial with an error rate lower than 10% [40] within minutes 2–4 of the anticipation phase. Heart rate and heart rate variability parameters were then extracted for these 1 min intervals with low error rates. We extracted heart rate (HR), the root mean square of successive interbeat interval differences (RMSSD) as an indicator of parasympathetic activity, and sympatho-vagal balance. Sympatho-vagal balance was expressed as sddn/RMSSD, with sddn being the standard deviation of all the interbeat intervals.

#### 2.5.2. General Movement Activity

An MSR145 mini Data Logger (MSR Electronics GmbH, Seuzach, Switzerland) measured the piglets’ general movement activity non-invasively. The logger was fixed with a self-adhesive, elastic bandage (equiLASTIC, EUROFARM GMBH, Bützberg, Switzerland) on the right hind leg of each piglet. During the trial, changes in acceleration of the x, y, and z-acceleration axis of the left hind leg were registered once a second and stored. The y- acceleration axis ran parallel to the longitudinal axis of the piglets’ hind leg. The absolute values of the acceleration changes of the x, y, and z- axes were summed across minutes 2–4 of the anticipation phase.

After a day’s trials, the data were transferred to a computer via an USB interface and exported as a csv (MSR Reader, version 5.24.02; MSR Electronics GmbH, Seuzach, Switzerland) for further analysis. The MSR logger was set to log a maximum of twofold force of gravity (2 g) for the acceleration values. This maximum value was reached once in four trials (0.9615% of all trials). The computer on which the programs Polar Team^2^ and MSR Reader ran was time-synchronised with the watch that was used to record the start time of the trials, such that the data of the trials could be identified.

### 2.6. Statistics

#### 2.6.1. Sample Size

In principle, 960 (2 piglets × 32 pairs × 3 valences × 5 days) observations would have been possible for both heart rate and activity data. In 124 trials, the data of both piglets in a pair did not reach error rates that were lower than 10% for the heart rate measures. In an additional 203 trials, only the data from one piglet were available resulting in 509 observations (960 − 2 × 124 − 203). In 327 of these 509 observations, a second minute was available with less than 10% error rate (resulting in a total number of observations of 836 = 509 + 327). 23 of the 960 potential data points for the activity measure were unusable because the MSR logger fell off during the trial or was set up incorrectly, resulting in a total of 937 observations. In addition, five outliers in the heart rate (0.6%) and five outliers in the activity data (0.5%) as visible in the residual plots were omitted from analysis. In one case, it was noted that one piglet was sick (slight case of diarrhea) and nine piglets showed extensive activity and vocalisations. Mostly, these behaviours occurred due to a fight between piglets during the anticipation phase and therefore a specific reaction to the experimental condition seemed unlikely. Therefore, the final number of observations that were included in the analysis was 821 (836 − 5 − 10) and 922 (937 − 5 − 10) for the heart rate and the activity data, respectively.

#### 2.6.2. General Aspects of the Statistical Models

We used linear mixed effects models implemented with the function lmer (package: lme4, [41]) in R (version 3.2.1 for the preliminary and 3.4.3 for the final evaluation [42]).

We checked statistical assumptions, the distribution of the errors and random effects, as well as the homogeneity of the errors using a graphical analysis of residuals.

*p*-values were calculated based on a parametric bootstrap approach (package pbkrtest [43]). In the final evaluation, we calculated a full-model using sum-contrasts of the fixed effects. An overall *p*-value was then calculated when comparing the full with the null-model. If this comparison reached a relatively low *p*-value (<0.05), we calculated *p*-values for the single effects in the model by comparing the full model with the model omitting the corresponding effect. Using sum-contrasts allowed for the interpretation of main effects, even in the presence of statistical interactions (full model analysis [44]).

#### 2.6.3. Preliminary Evaluation

We used activity, heart rate, heart rate corrected for activity (expressed also as a ratio [45]), and heart rate variability (RMSSD and sdnn/RMSSD) as outcome variables in the preliminary evaluation. The ratio of heart-rate corrected for activity was used to account for the fact that increased activity alone can change heart-rate parameters [45]. Using a log transformation for all the outcome variables resulted in errors and random effects that were normally distributed. These outcome variables were used in dependence of the fixed effects valence (factor with three levels: negative, intermediate, positive), number of repetition of each stimulus during a week (factor with five levels), and the week (factor with three levels reflecting the order of testing) and all of their interactions. The random effects hierarchically nested week within piglet identity, within pair identity, and within batch identity. A crossed-random effect of trial identity nested within date corrected for the fact that the piglets in a pair were always tested simultaneously and that the trials of a given batch were conducted on the same day. To account for the variability between the different stimuli within a given valence, an additional crossed random effect was included for the interaction of valence (fixed) and treatment (random).

As we focused on the anticipation phase, the first trial in each week could be expected to differ from all the other trials of the same week, because piglets had not yet experienced the stimulus with which they were about to be confronted. In addition, in weeks 2 and 3, a carry-over effect could have been expected due to the piglet’s prior experiences. Yet, we did not find an interaction of week and valence, which could have indicated such a carry-over effect. Neither could we observe that the variability in the piglet’s reaction was larger on the first day in weeks 2 and 3, which could have been expected due to the fact that piglets had different prior experiences. We therefore concluded that the two days between the weeks were sufficient to mitigate carry-over effects and that the first day of a given week could be treated as a baseline for the week. This led us to the approach used in the final evaluation.

#### 2.6.4. Final Evaluation

In the final evaluation, we expressed the piglet’s reactions on days 2–5 relative to their baseline on day 1 in each week (we used a ratio to do so). We did so for the general movement activity, heart rate, heart rate corrected for activity (expressed also as a ratio [45]), RMSSD, and sdnn/RMSSD.

With the heart rate data, on each day, one or two observations were available. If for a given day (2–5) only one observation was available, this value was divided by either the single value of day 1 or one randomly chosen value if two were available on day 1. If for a given day (2–5) two observations were available, they were either both divided by the value for day 1 or the two values of day 1 were randomly assigned to the two values of the given day. This was done rather than using an average of the baseline, such that the precision of all our observations was kept at the level of the raw data (whereas a mean value would have had a higher precision).

To be able to calculate the relative changes on days 2–5, a measurement on a given day as well as one on day 1 was necessary. For the heart rate corrected for the activity, all four measurements were necessary (heart rate and activity, on a given days 2–5 and on day 1). The number of observations that could be evaluated was therefore 737, 382, as well as 371 for activity, for HR, RMSSD, and sympatho-vagal balance, as well as the heart rate corrected for activity. This data is provided online as Supplementary Materials.

The fixed effects were the presumed valence of the stimuli (factor with three levels: negative, intermediate, positive), number of repetition of each stimulus during a week (factor with four levels), and the week (factor with three levels reflecting the order of testing) and all of their interactions.

Apart from the interaction between the fixed effect valence and the random effect treatment, the random effect of the preliminary evaluation was adopted. The interaction was dropped because, in the data structure of the final evaluation, non-convergences occurred often in the estimation of the model and the parametric bootstrap that was used for the calculation of *p*-values. Moreover, several random effects were estimated to be zero. Both these problems indicated that the model was over-specified.

## 3. Results

**Figure 3:**
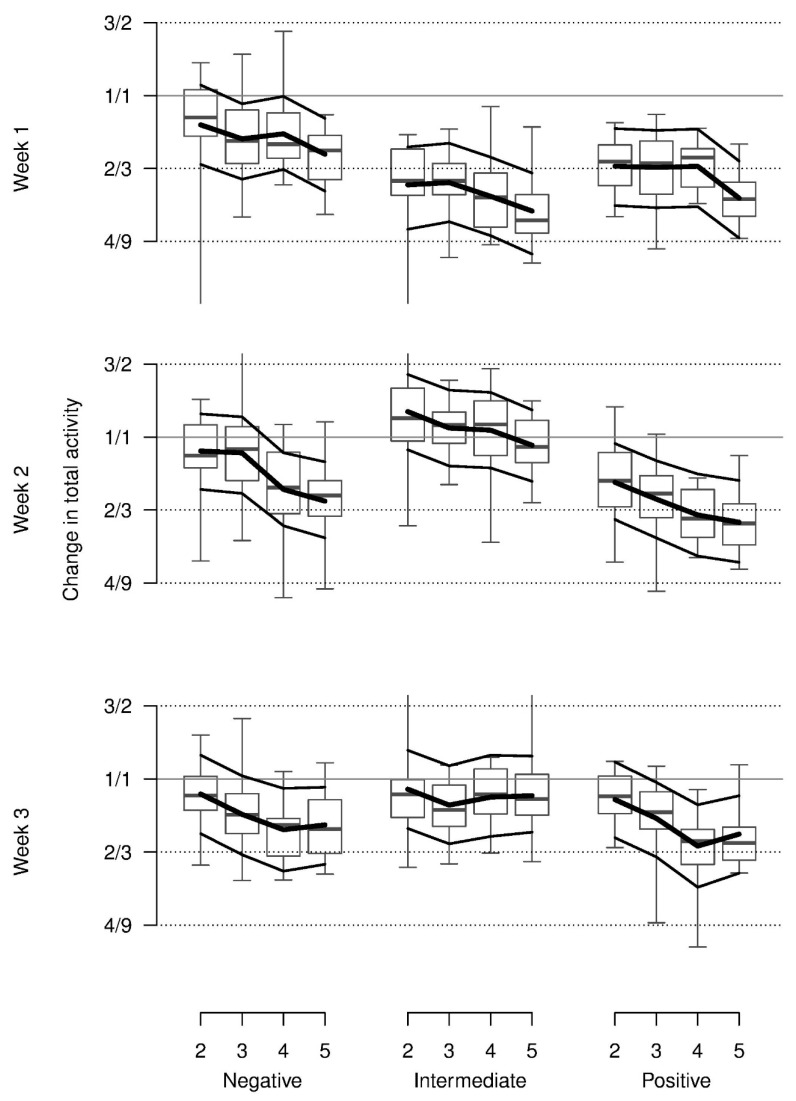
Relative total activity of piglets during the anticipation phase on days 2 to 5 in comparison with the first day in each of the three weeks (baseline) when confronted with negatively, intermediate and positively valenced stimuli. Y-axis is on a log scale with labels on the original scale. Boxplots show the medians, quartiles and data range. Thick lines: model estimates, thin lines: upper and lower 95% confidence interval based on a parametric bootstrap.

On average, relative total activity was lower with the presumed positive stimuli than with the negative or neutral stimuli and lower in the first as compared to the second and third week. In addition, this pattern was modulated by an interaction indicating more activity in the negative condition in week 1 and increasing activity from the positive to the negative and neutral condition in weeks 2 and 3. Additionally, relative total activity decreased across repetitions for all stimuli and weeks (Table 1, Figure 3). Almost all of these values were lower than during baseline.

**Figure 4:**
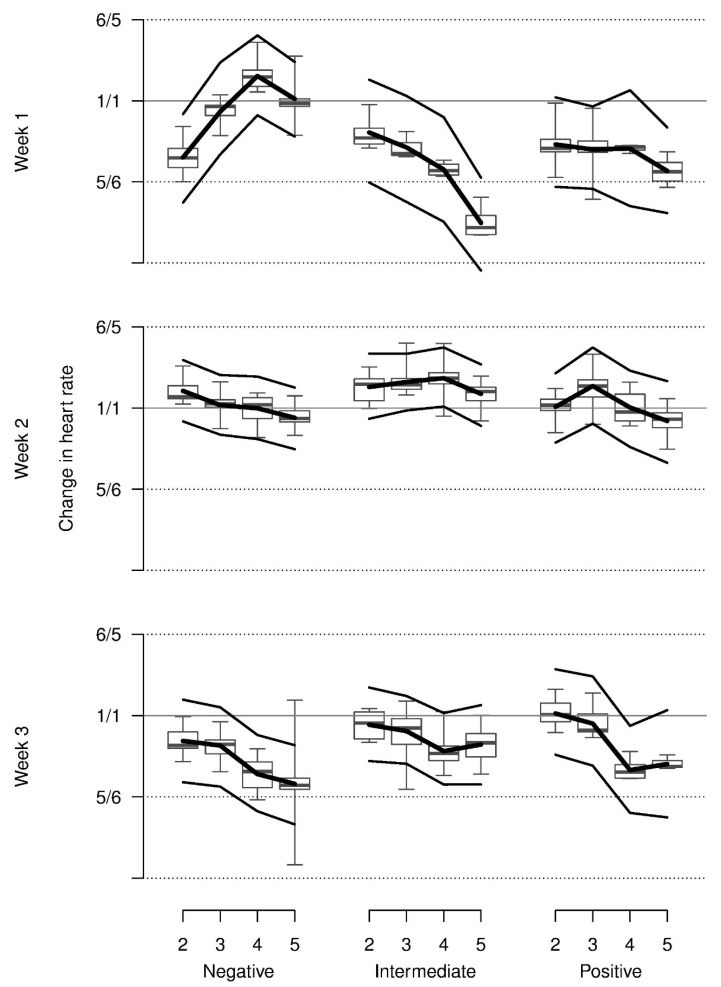
Relative heart rate of piglets during the anticipation phase on days 2 to 5 in comparison with the first day in each of three weeks (baseline) when confronted with negatively, intermediate and positively valenced stimuli. Y-axis is on a log scale with labels on the original scale. Boxplots show the medians, quartiles and data range. Thick lines: model estimates, thin lines: upper and lower 95% confidence interval based on a parametric bootstrap.

Relative heart rate decreased from repetition 2 onwards on average and was higher in week 2 when compared with weeks 1 and 3. Most observed values were somewhat lower than those that were shown at baseline. The increase in heart rate across repetitions for the negative stimulus in week 1 could only marginally be supported statistically (three-way interaction, Table 1, Figure 4). 

**Figure 5:**
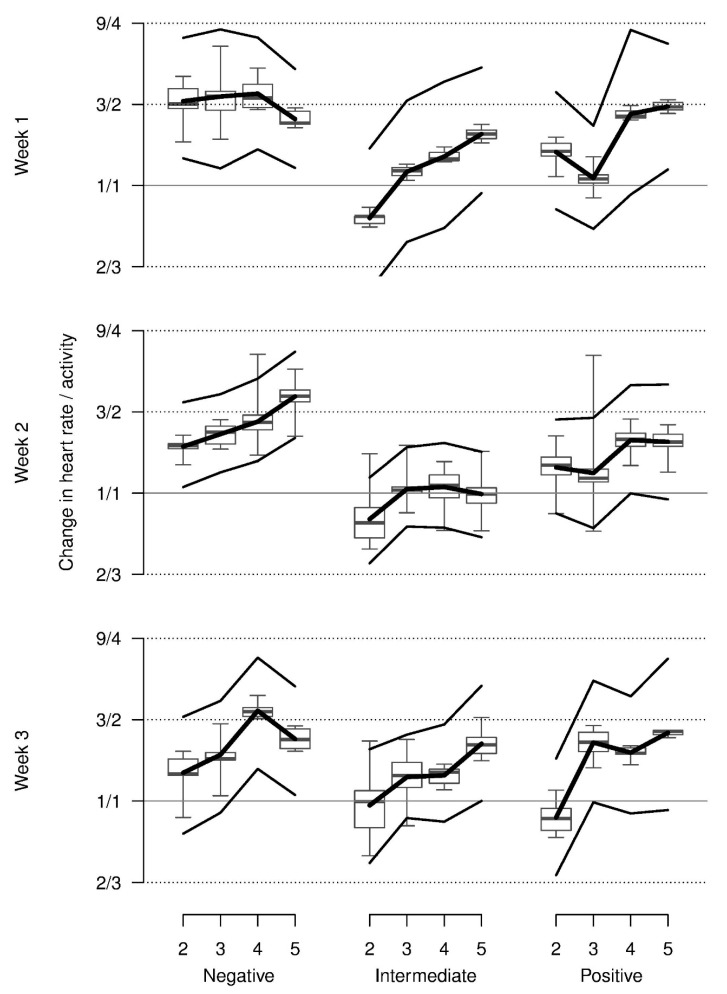
Relative change in heart rate corrected for activity of piglets during the anticipation phase on days 2 to 5 in comparison with the first day in each of the three weeks (baseline) when confronted with negatively, intermediate and positively valenced stimuli. Y-axis is on a log scale with labels on the original scale. Boxplots show the medians, quartiles, and data range. Thick lines: model estimates, thin lines: upper and lower 95% confidence interval based on a parametric bootstrap.

Though relative heart rate corrected for activity seemed to increase across repetitions for most stimuli and weeks starting at the approximate baseline value and seemed elevated for the negative stimuli in week 1, this could not be supported statistically (Table 1, Figure 5). No statistical support of systematic effects of valence, repetition, and week on RMSSD, and sympatho-vagal balance could be found and no systematic patterns were visible in the model estimates either (not shown, Table 1).

The behaviour of the piglets when confronted with the individual stimuli was not directly comparable in this study due to the diversity of stimuli used. Therefore, it was not quantified. Nevertheless, a brief qualitative description may help to interpret the quantitative data from the anticipation phase. When piglet pairs were exposed to negatively valenced stimuli, they often vocalised and constantly moved around the test pen. When isolated, the piglets raised themselves on the walls or manipulated the door. Popping balloons and rattling plastic bags elicited a flight reaction in the piglets. When the piglet pairs were confronted with an inaccessible feed, they bit the Plexiglass box, stepped on it, turned it around, or kicked it away. The intermediate stimuli triggered very few reactions. The piglets remained calm and vocalised very little. The interest waned rapidly and the piglets were then standing close to the exit door until the trial was over. With what were presumed to be positively valenced stimuli, piglets were similarly calm and vocalised as little as with the intermediate stimuli. The time that was spent exploring decreased slowly from trial to trial.

## 4. Discussion

We set out to test the hypothesis that primary (innate) negative stimuli lead to a faster habituation than secondary (learnt) positive stimuli [1,4]. We found no consistent effect of an interaction between valence of the stimuli and the repetition, though that would point towards such a pattern. There was no clear main effect of valence, either. Therefore, we could not support this hypothesis. The assumption of our negative and positive stimuli being primary and secondary, respectively, may have been wrong. This would lead us back to the original hypothesis based purely on the asymmetry of fitness consequences [1] that implies a faster habituation for the positive when compared with the negative stimuli. Yet, this hypothesis could not be supported either. Finally, the patterns that we observed did not point towards a strong change from baseline without habituation. Several aspects could have caused this picture: (1) stimuli may not have differed in valence or not in the way that was presumed; (2) the experimental design may not have been adequate to test the hypothesis; or, (3) measures were not suitable etho-physiological indicators for detecting habituation. These three aspects will now shortly be discussed in turn.

We used stimuli that seem to share a wide consensus in respect to their valence (see introduction). Moreover, the reactions of the piglets when directly confronted with the stimuli seemed to be consistent with our presumed valence. Even though the presumed intermediate and positive stimuli shared some characteristics, they did not clearly group together in our analyses. Nevertheless, the variety of stimuli used here may have led to additional variability in the data, indicating that aspects other than valence were more prominent to the piglets. Only few stimuli have indeed been tested in respect to their valence and it seems that this task is more difficult than one could naïvely assume ([45], this volume). More effort should therefore be put in establishing the valence of stimuli in an independent way before using them in experiments.

We think that we have used stimuli that are quite strong. Nevertheless, the experimental situation in which piglets were taken from their group, equipped with the measurement devices and confronted with stimuli in a test pen could potentially have overshadowed the experimental treatments. Moreover, testing the piglets shortly after weaning and re-grouping could have led to a ceiling effect. This seems unlikely because they reacted specifically to the different stimuli presented. Strong carry-over effects might also have hidden the relevant patterns. If the latter was the case, we could have expected some changes across the weeks, but this was not observed. Also, given that the piglets only needed little driving between the home and test pen indicates that the overall situation of the experiment was not strongly aversive. Indeed, the pattern towards the negative stimuli in week one were suggestive even though they could not be clearly supported statistically: total activity was elevated, heart rate increased such that a high and constant level of activity-corrected heart rate was observed. This would indicate a strong reaction without habituation. In this sense, there could have been a lack of statistical power given a relatively large variability in the data. At least for the negative stimuli, this would be contrary to the theoretical predictions where a high level of consistency in response to a primary negative stimulus could be expected. There was some confounding of session length with valence of the stimuli in that negative sessions were more often shortened. This may have made the negative stimuli less severe from the point of view of the piglets. Finally, our piglets may not have learnt the association between the start box and the subsequent stimuli. We do not have independent support that they did in our current experiment, but the piglets seem to be able to make such associations in similar experiments [34].

We applied wide-spread indicator variables in an anticipation phase to measure the piglets’ reactions. The fact that we hardly saw any sign of carry-over effects might indicate that these indicators were not sensitive enough, though similar approaches have been used previously (e.g., [24,34]). Nevertheless, a comparison of the direct reaction to the stimuli may be desirable, although the use of indicator variables may be more difficult then, given the fact that a variety of stimuli were used. Other approaches such as place conditioning [7] or operant procedures ([46], this volume) may then be needed.

The only effect that was apparent with several outcome variables (activity, heart-rate, heart rate corrected by activity) was a change in the relative reactions with repetitions within each of the weeks. It therefore seems that the piglets did habituate to an aspect within the weeks but not across weeks. Perhaps, this was due to the novelty of each stimulus that the piglets encountered in the different weeks [2].

## 5. Conclusions

In conclusion, we think that there was no obvious problem in our experimental design or in all of our measures. We therefore conclude that we need to improve our knowledge on which stimuli are considered negative and positive from the point of view of the animals. Based on the stimuli used here, we could not support the notion that the habituation process differs according to the valence of the stimuli. Other aspects of the stimuli may have been more important in respect to the habituation process.

## Figures and Tables

**Table 1:** *p*-values (parametric bootstrap) for the different outcome variables. A maximum model was compared with the null model (“global” *p*-value) and then with a model from which the given variable was excluded (using sum-contrasts for the predictor variables).

Predictor Variables	Activity	Heart Rate	Heart Rate/Activity	RMSSD	sdnn/RMSSD (Sympatho-Vagal Balance)
All (global *p*-value)	0.002	0.003	0.29	0.69	0.21
Main effects:					
Valence	0.04	0.86	-	-	-
Repetition	0.001	0.01	-	-	-
Week	0.003	0.001	-	-	-
Interactions:					
valence × repetition	0.95	0.46	-	-	-
valence × week	0.03	0.13	-	-	-
repetition × week	0.54	0.25	-	-	-
valence x repetition × week	0.98	0.10	-	-	-

RMSSD: root mean square of successive interbeat interval differences; sdnn: standard deviation of all interbeat (NN) intervals.

## Supplementary Materials

The following are available online at http://www.mdpi.com/2076-2615/8/9/149/s1.
